# Supplementary material for: A substrate-multiplexed platform for profiling enzymatic potential of plant family 1 glycosyltransferases
Source: Nat Commun. 2025 Jul 10;16:6366. doi: 10.1038/s41467-025-61530-6 (PMC12246196; doi:10.1038/s41467-025-61530-6)
Supplement: Supplementary file 1 — Supplementary Information [file 41467_2025_61530_MOESM1_ESM.pdf]

A substrate-multiplexed platform for profiling enzymatic potential of plant family 1 glycosyltransferases

Sirirungruang and Blay *et al.*

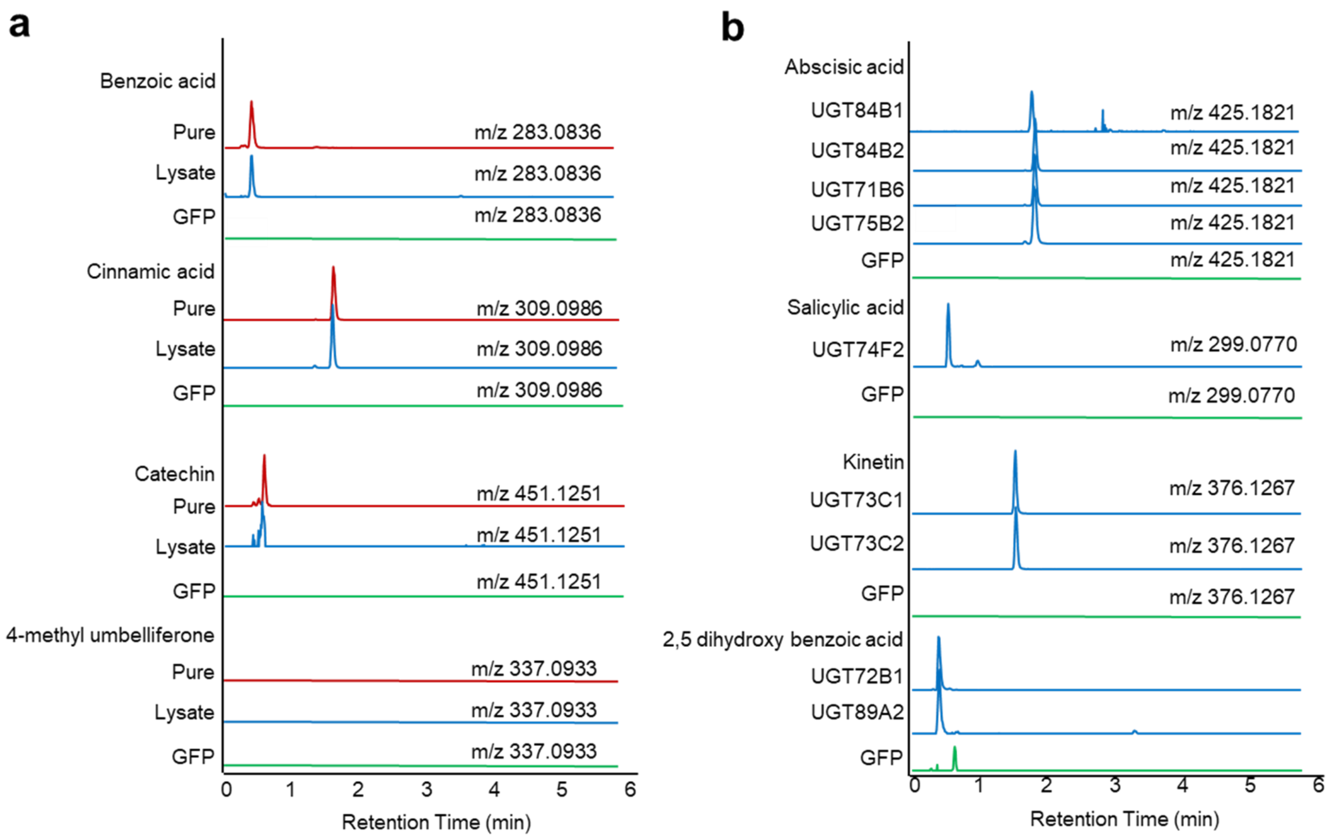

Supplementary Figure 1. Glycosylation reactions by clarified *E. coli* lysate expressing plant family 1 GT enzymes. a) Extracted ion chromatograms of glycosylation reactions by purified UGT75B1 (red), in comparison to clarified *E. coli* lysate expressing UGT75B1 (blue) or GFP (green). Benzoic acid, cinnamic acid, and catechin glycosylation results indicate that the clarified lysate of *E. coli* expressing the GT enzyme has glycosylation activity comparable to purified enzyme[12], and that the activity is absent in *E. coli* expressing GFP. 4-methylumbelliferone serves as a negative control as it is not a substrate of UGT75B1. b) Extracted ion chromatograms of previously reported glycosylation reactions[40,62,63] by clarified *E. coli* lysate expressing various family 1 GT enzymes (blue) in comparison to GFP (green). Results indicate that glycosylation activity by *E. coli* lysate is generalizable among plant family 1 GT enzymes.

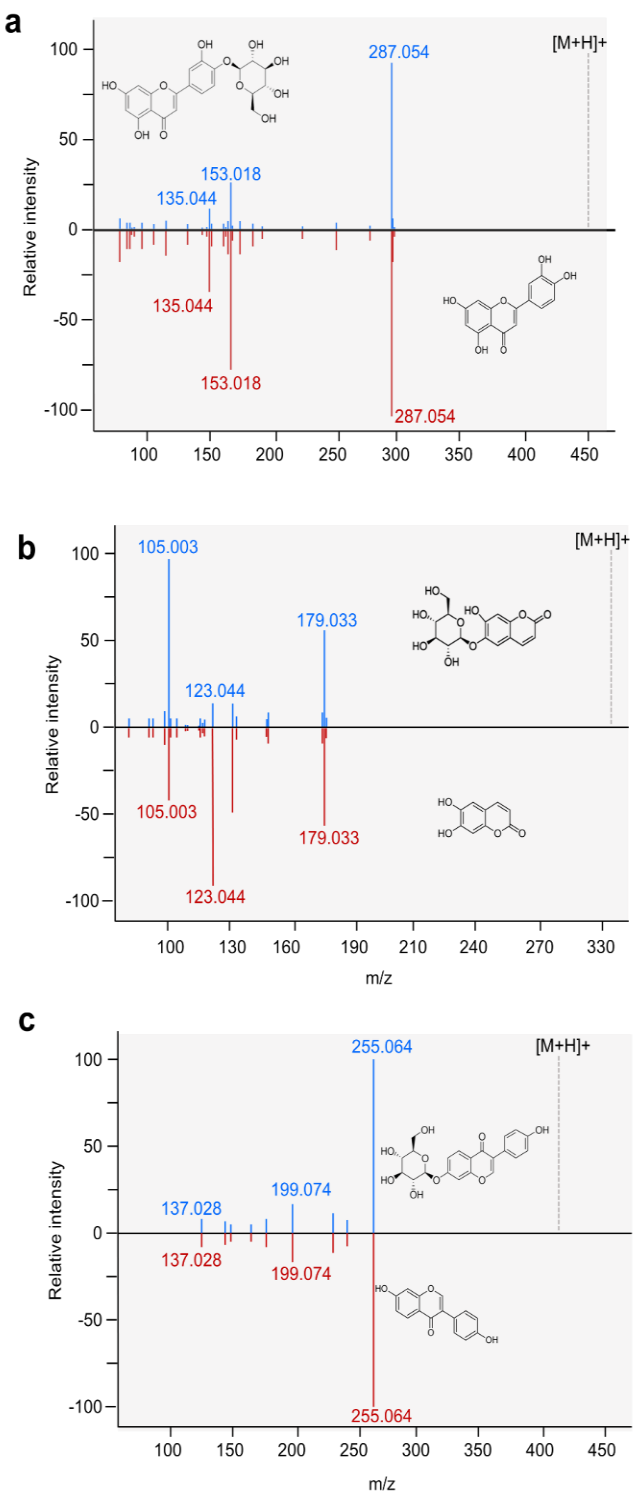

Supplementary Figure 2. Mirror plots comparing the MS/MS spectra of authentic standards of glycosides and corresponding aglycones. Spectra of glycosides (blue, top) were used to query the database, and their top matches (red, bottom) correspond to their aglycone counterparts. MS/MS spectra of luteolin-4'-*O*-glucoside (a), esculetin-7-*O*-glucoside (b), and daidzein-7-*O*-glucoside (c) match those of luteolin, esculetin, and daidzein.

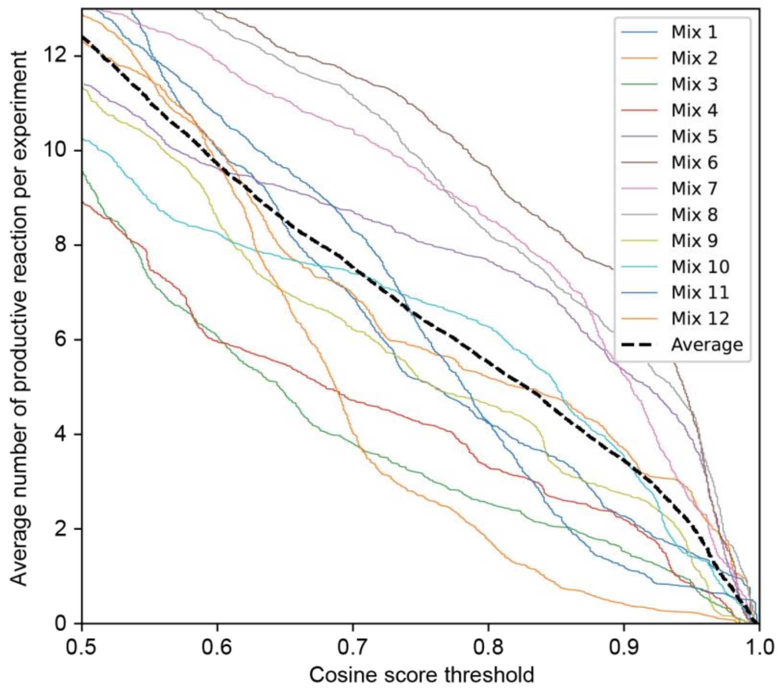

Supplementary Figure 3. Effects of cosine score threshold on the number of glycosylation reactions classified as productive. The number of productive reactions per experiment was calculated for each enzyme and each mix of 40 substrates using cosine score thresholds from 0.5 to 1.0. At each threshold value, the number of productive reactions was then averaged over all enzymes for each mix of substrates (solid color lines). Values of all mixes were then averaged together to yield the average for the entire lysate screen (dashed black line). The number of productive reactions increases at a high rate at high cosine scores (>0.9) before the rate of increase drops slightly to a steady slope at lower cosine scores (<0.9).

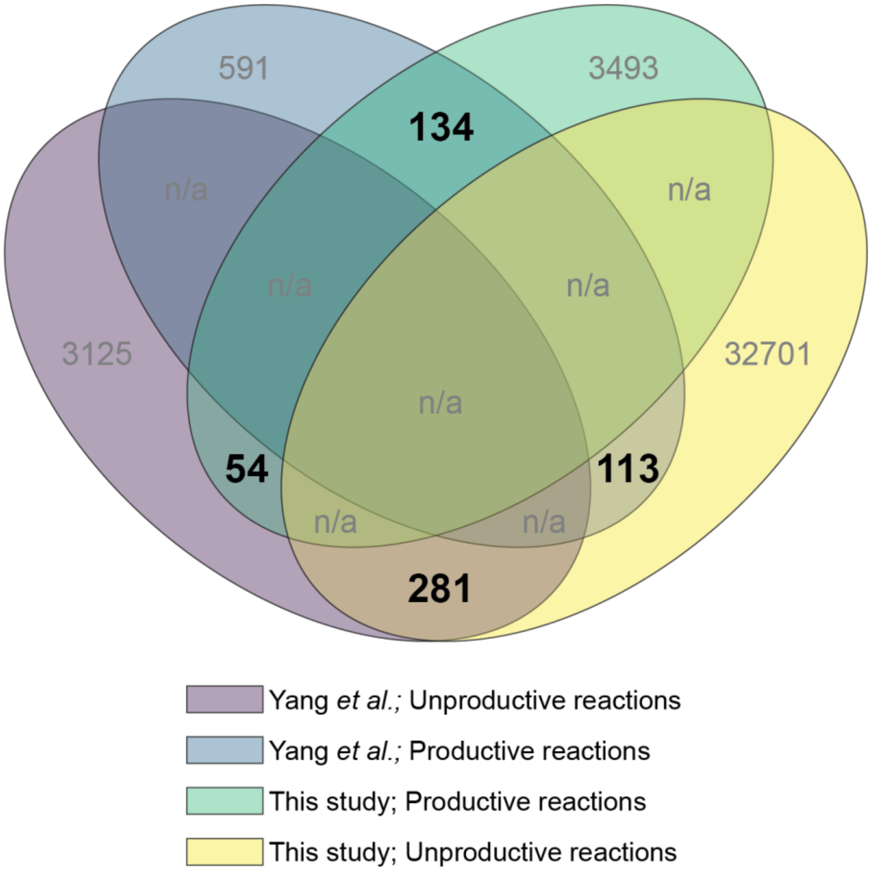

Supplementary Figure 4. Comparison of glycosylation reaction outcomes between lysate-based screen and Yang *et al.* A total of 582 glycosylation reactions concerning 17 sugar acceptor substrates and 36 enzymes were common between the two studies with regards to enzyme and substrate identity. However, the two studies differ in experimental conditions. While Yang *et al*. performed glycosylation reactions at pH 7.8 with 177 µM of UDP-glucose and 0.1 mg/mL of substrates, the lysate screen was carried out at pH 6.8 with 83 µM of UDP-glucose and 10 µM of substrates. Despite being performed under different reaction conditions and analysis methods, the two studies agreed on 415 reactions or approximately 70% of outcomes. Numbers representing the reactions common between the two studies are shown in black and bolded while all other numbers are shown in grey.

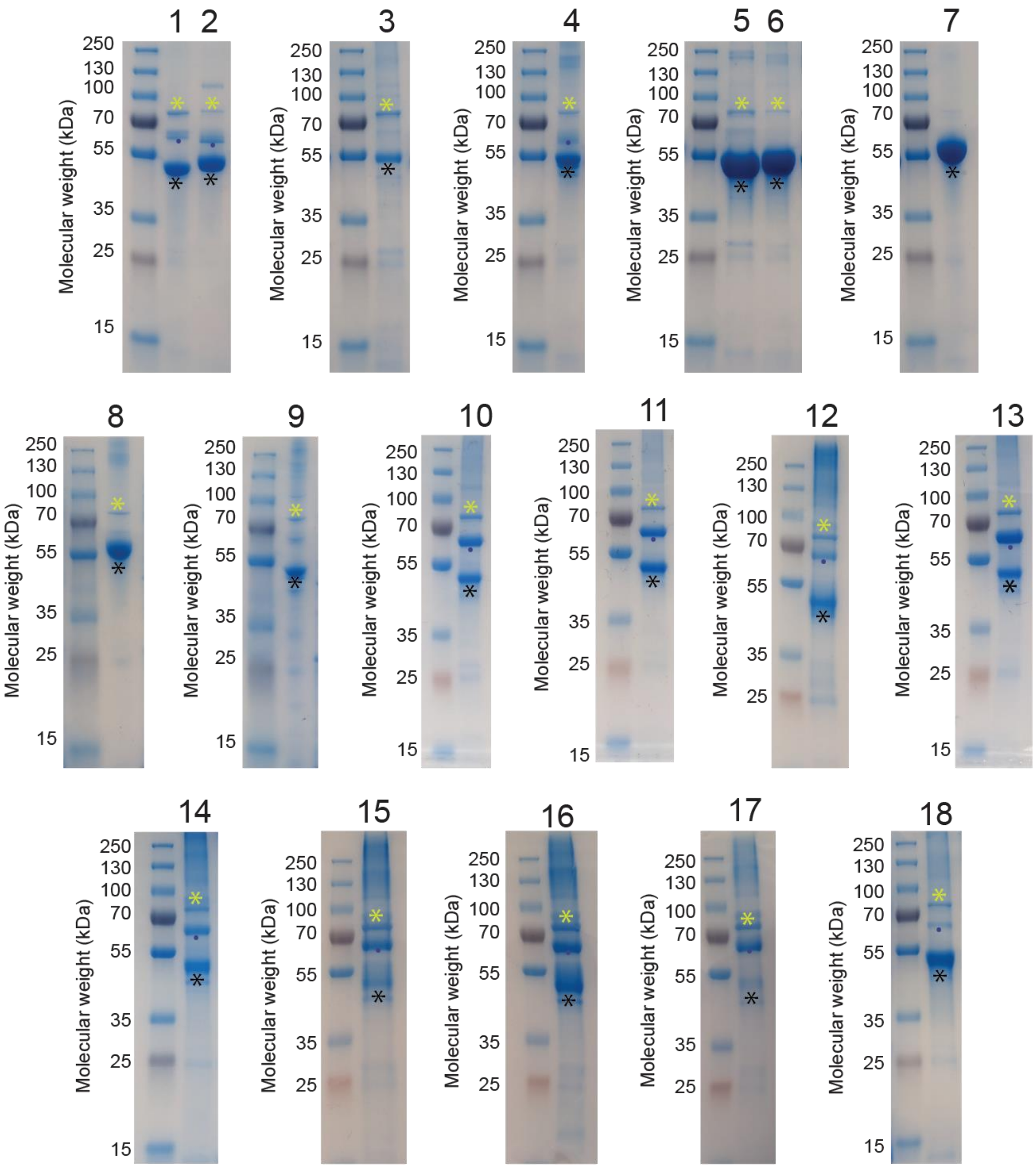

Supplementary Figure 5. SDS-PAGE analysis of purified protein used in this study. Lane 1: UGT75B2; Lane 2: UGT71C4; Lane 3: UGT75D1; Lane 4: UGT72B2; Lane 5: UGT72B1; Lane 6: UGT74D1; Lane 7: UGT73C5; Lane 8: UGT73C4; Lane 9: UGT87A2; Lane 10: UGT76C2; Lane 11: UGT76C2 C20H; Lane 12: UGT76C2 C20A; Lane 13: UGT76C2 D112A; Lane 14: UGT76C3; Lane 15: UGT76C3 C19H; Lane 16: UGT76C3 C10A; Lane 17: UGT76C3 D117A; Lane 18: UGT76C5.Black asterisks, black dots, and yellow asterisks indicate target glycosyltransferase proteins, uncut GST-glycosyltransferase fusions, and the common contaminant GroEL, respectively.

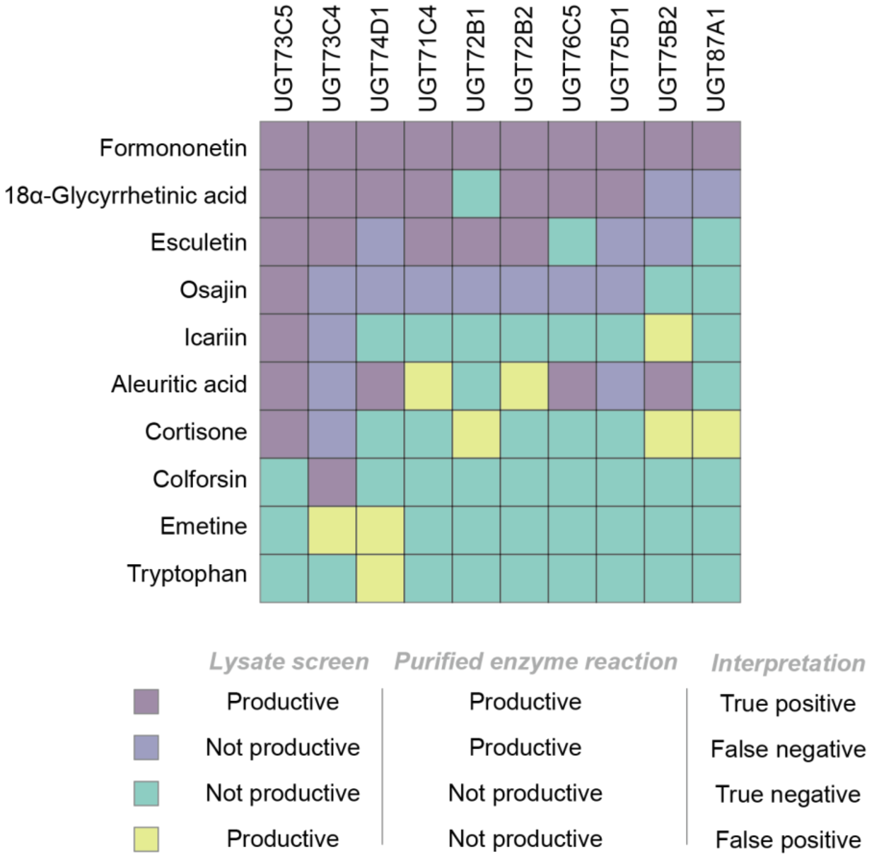

Supplementary Figure 6. Comparison of glycosylation reaction outcomes between lysate-based screen and purified enzyme reactions. One hundred reactions concerning ten enzymes and ten sugar acceptor substrates were validated using purified enzymes without substrate multiplexing. Purified enzyme reactions were performed using 500 µM UDP-glucose, 50 µM substrate, and 10 µM enzyme at pH 7.6, and their outcomes were taken as true reaction outcomes. Of 100 validated reactions, 30 were true positives (purple); 16 were false negatives (blue); 45 were true negative (green); and nine were false positives (yellow). Thus, the lysate screen yielded accuracy, precision, recall, specificity, and F1 values of 75%, 77%, 65%, 83%, and 71% respectively. All data represent 2-3 technical replicates.

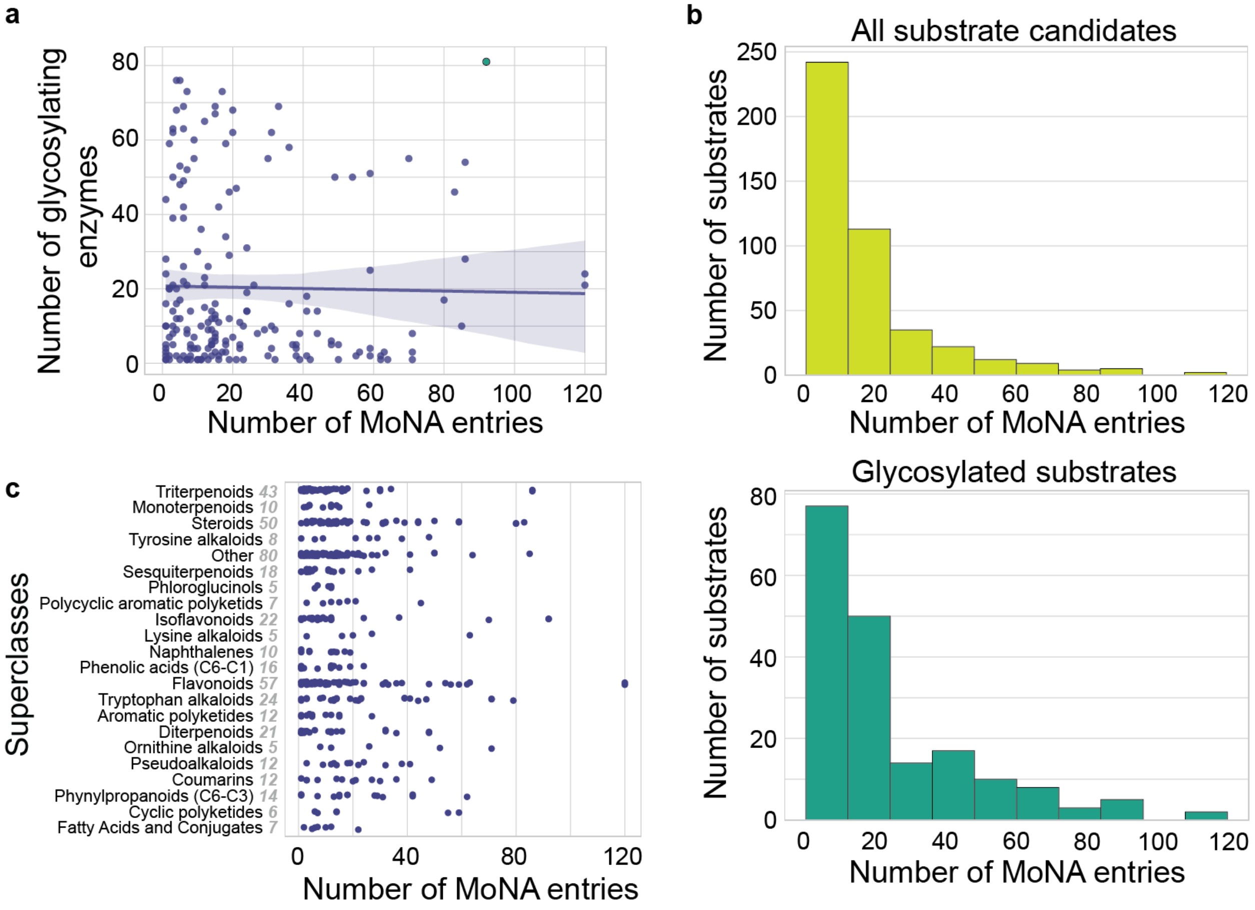

Supplementary Figure 7. Relationship between the number of reference spectra and likelihood of finding glycosylation products. a) No correlation was found when the number of MS/MS corresponding to each substrate molecule was plotted against the number of enzymes observed to glycosylate each molecule in the lysate screen. b) The distribution of all substrate candidates (yellow) and of glycosylation substrates (green) based on corresponding number of reference MoNA entries are similar, suggesting no bias on the likelihood of productive glycosylation reactions based on the number of reference MS/MS spectra. c) The distribution of each superclass of substrate candidates according to the number of reference MoNA entries shows no bias among superclasses.

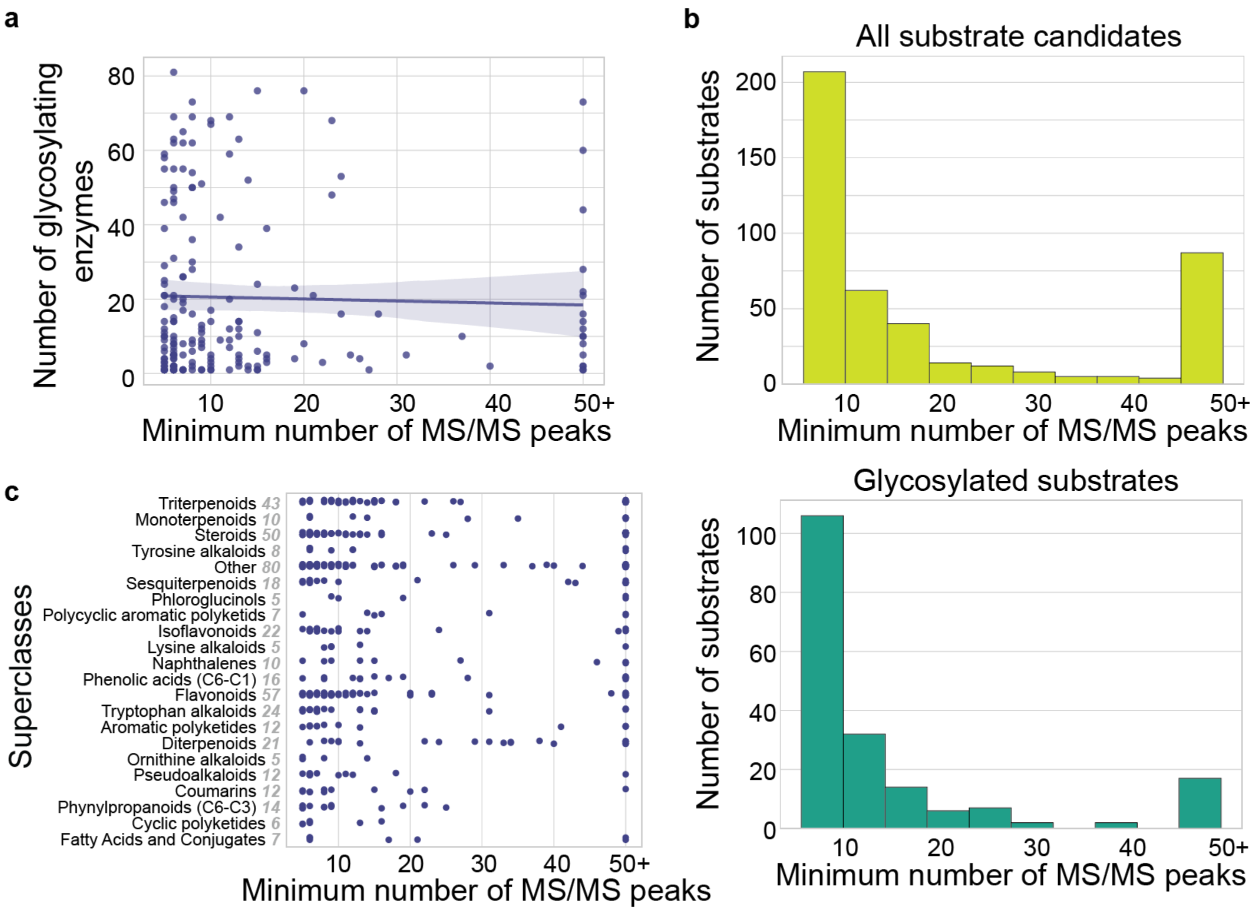

Supplementary Figure 8. Relationship between the minimum number of peaks in reference MS/MS spectra and likelihood of finding glycosylation products. a) No correlation was found when the minimum number of MS/MS reference spectral peaks corresponding to each substrate molecule was plotted against the number of enzymes observed to glycosylate each molecule in the lysate screen. b) The distribution of all substrate candidates (yellow) and of glycosylation substrates (green) based on the minimum number of MS/MS reference spectral peaks are similar, suggesting no bias in the likelihood of productive glycosylation reactions based on the minimum number of MS/MS reference spectral peaks. c) The distribution of each superclass of substrate candidates according to the minimum number of MS/MS reference spectral peaks shows no bias among superclasses.

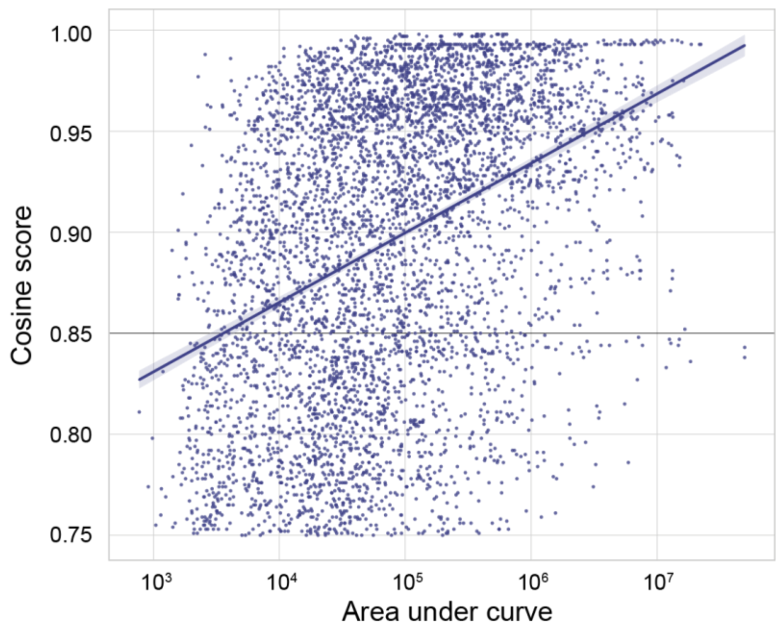

Supplementary Figure 9. Relationship between the signal intensity and likelihood of finding glycosylation products. The area under the curve in the MS1 channel was calculated for all mass entries from the lysate screen with a cosine score of 0.75 or higher and plotted against their cosine scores. A positive correlation was observed between signal intensity and cosine score. The correlation is conceivable as strong signals often yield higher quality MSMS spectra, resulting in better matching to reference spectra.

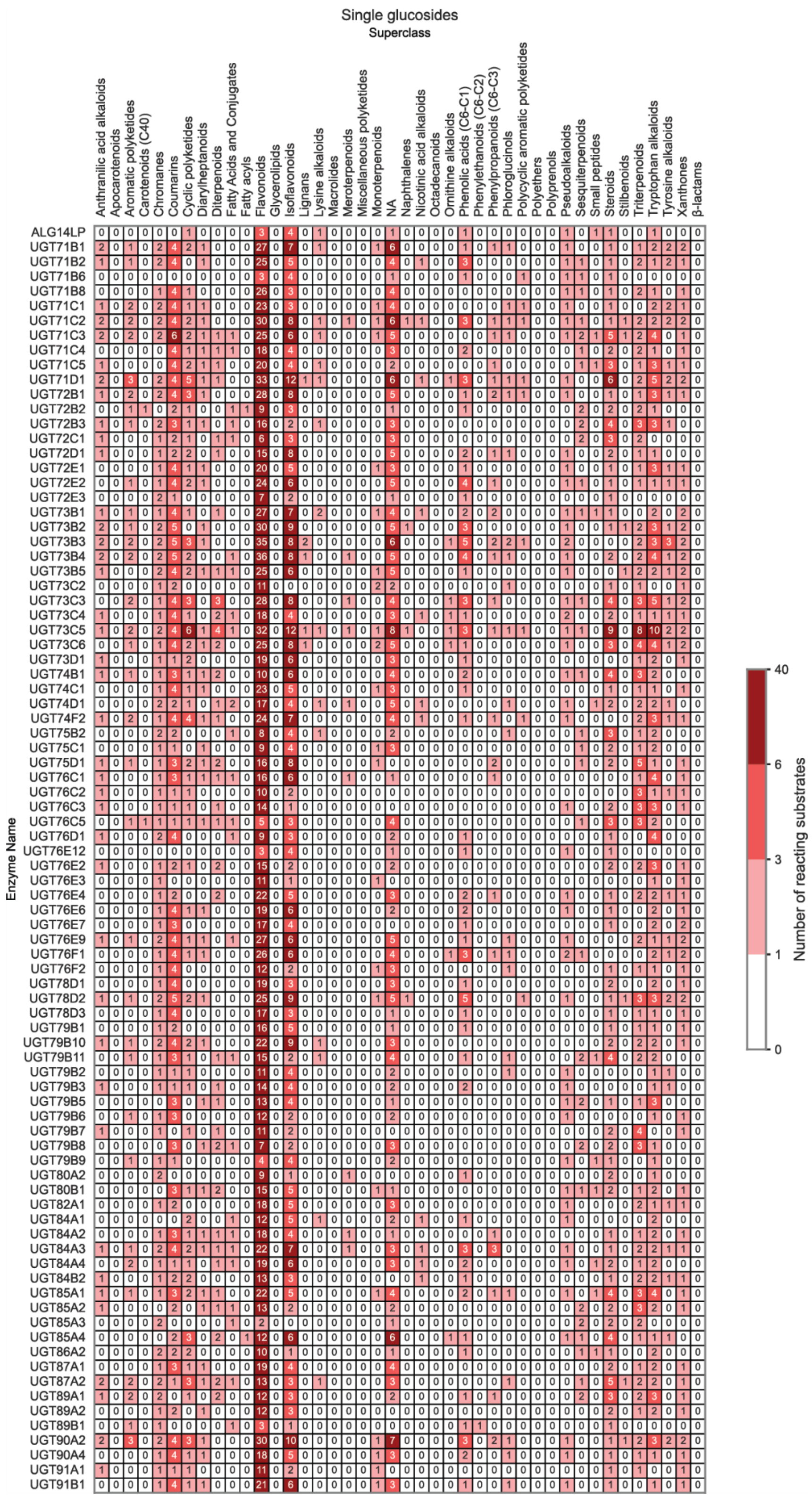

Supplementary Figure 10. Productive single glycosylation reactions identified by the large-scale lysate-based glycosylation platform. Pivot table shows the number of singly glycosylated products detected at cosine score threshold of 0.85 for each superclass of substrates by each enzyme. Enzyme names and substrate superclasses are ordered alphabetically.

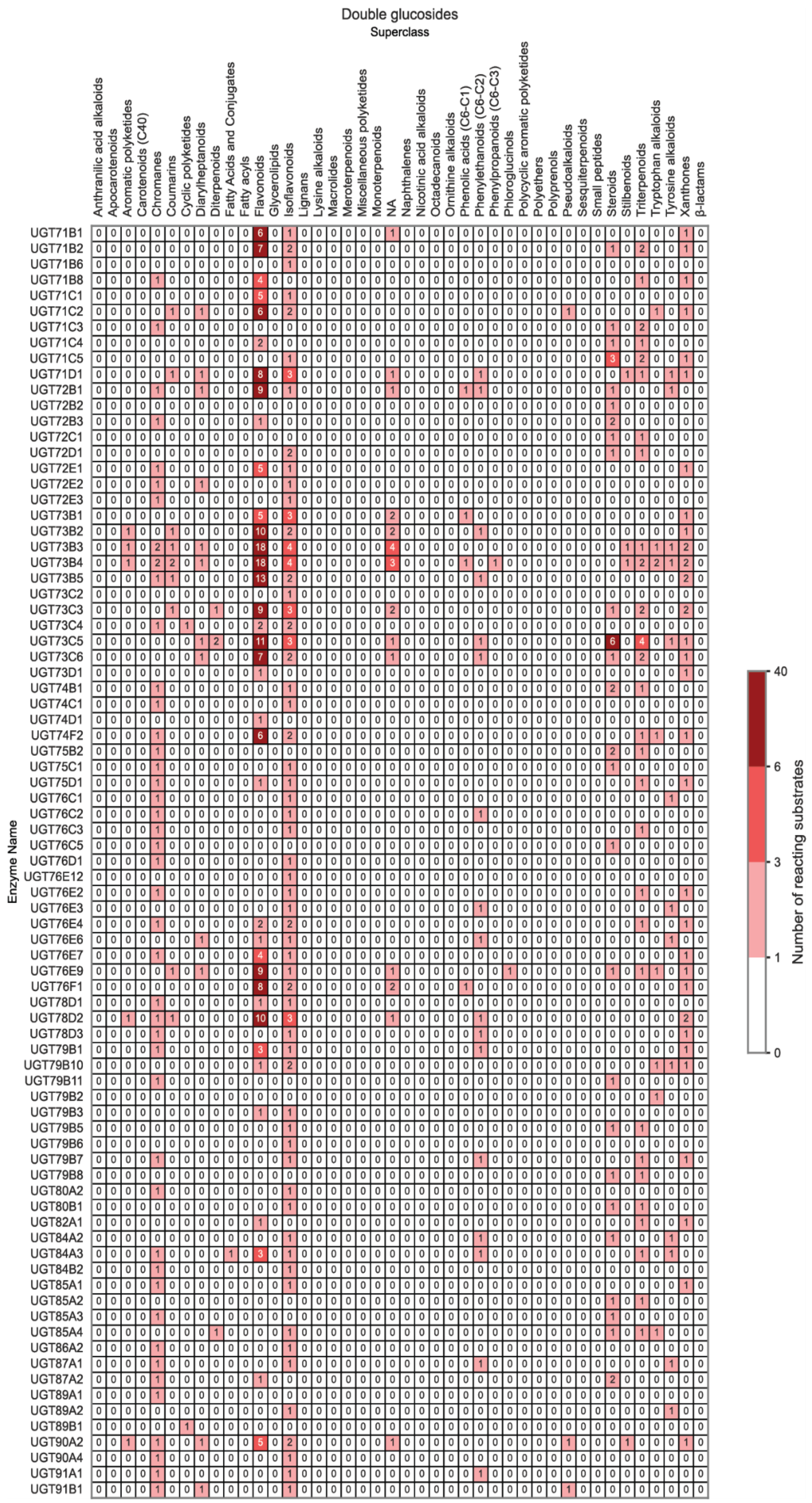

Supplementary Figure 11. Productive double glycosylation reactions identified by the large-scale lysate-based glycosylation platform. Pivot table shows the number of doubly glycosylated products detected at cosine score threshold of 0.85 for each superclass of substrates by each enzyme. Enzyme names and substrate superclasses are ordered alphabetically.

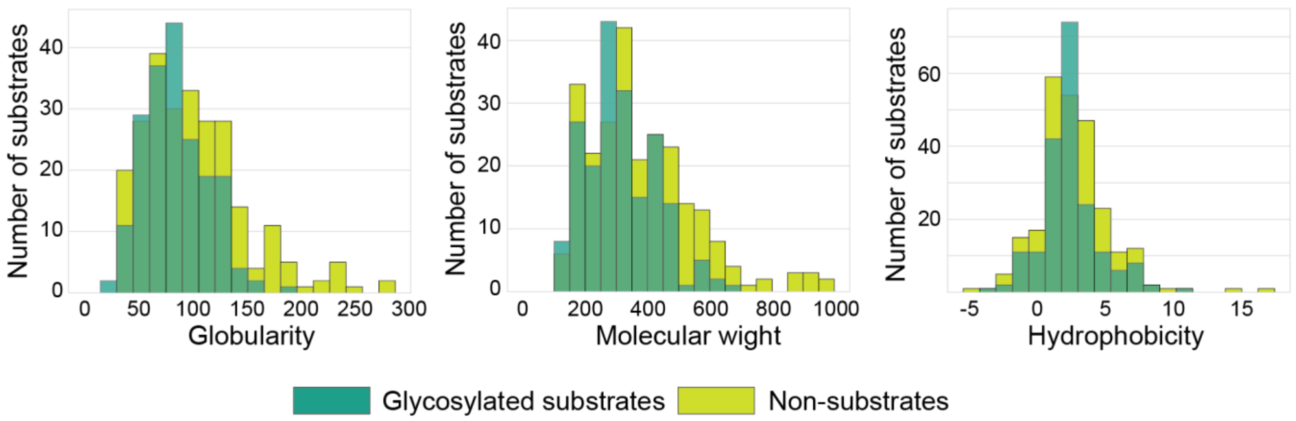

Supplementary Figure 12. Distribution of physical characteristics of substrates in comparison to non-substrates in the lysate screen. Histograms showing the distribution of substrates (green) and non-substrates (yellow) based on their globularity (left), molecular weight (middle), and hydrophobicity (right) show that family 1 GT exhibits a bias towards smaller and less globular molecules without regards to hydrophobicity.

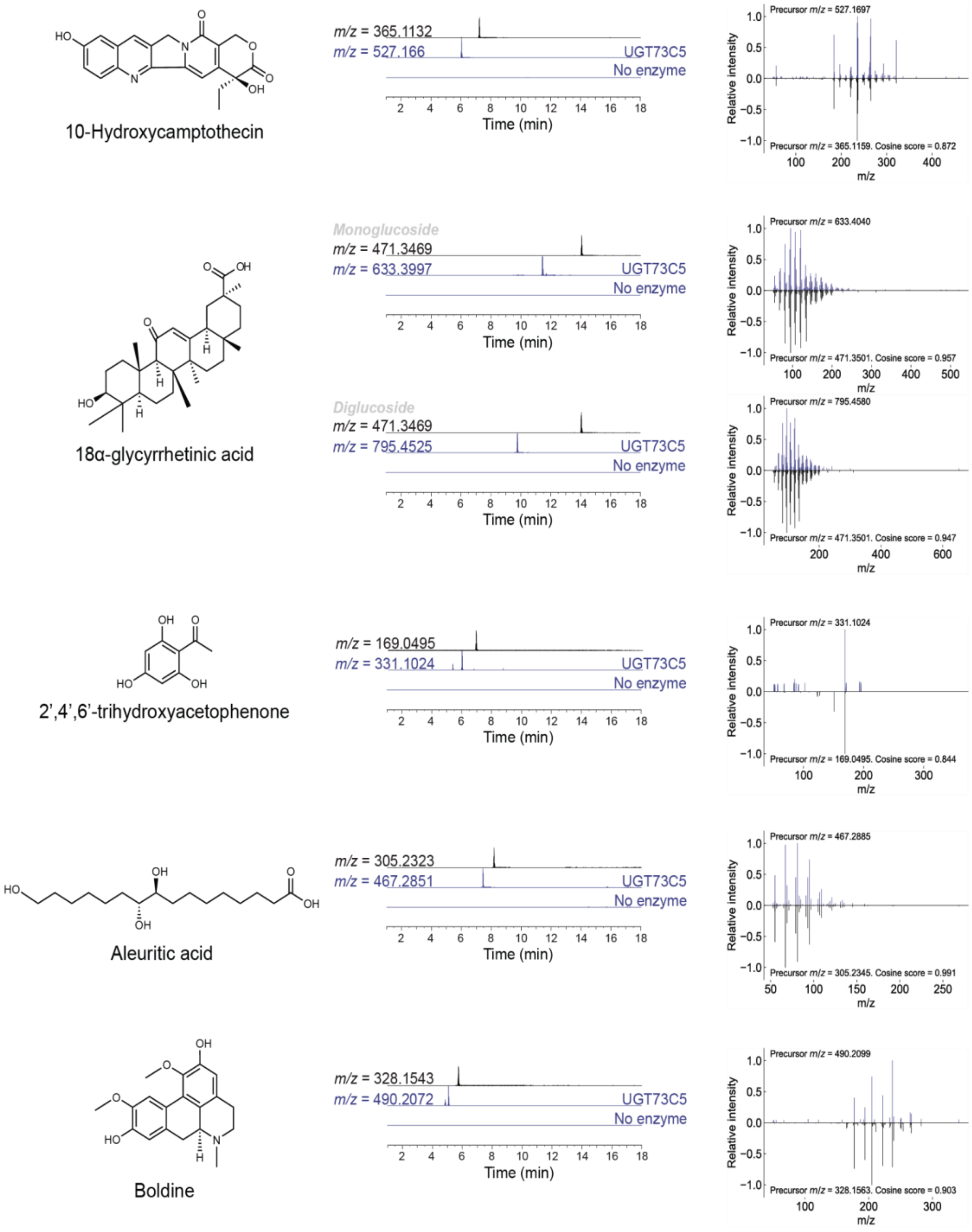

Supplementary Figure 13. Glycosylation products of 10-hydroxycamptothecin; 18α-glycyrrhetinic acid; 2', 4', 6'-trihydroxyacetophenone; aleuritic acid; and boldine by purified UGT73C5. For each substrate, extracted ion chromatograms of glycosylation products of reactions including purified UGT75C5 (purple, middle) or no-enzyme control (purple, bottom) are shown with *m/z* values corresponding to $[M+H]^+$ indicated to the left. Extracted ion chromatograms of substrates (black, top) show that glycosides' retention times shift forward in relation to their aglycone counterparts. A mirror plot showing the comparison between an experimental glucoside MSMS spectrum (purple, top) and its matching reference substrates spectrum (black, bottom), along with the corresponding cosine score, is shown next to each product chromatogram. All data are representative of at least 3 technical replicates.

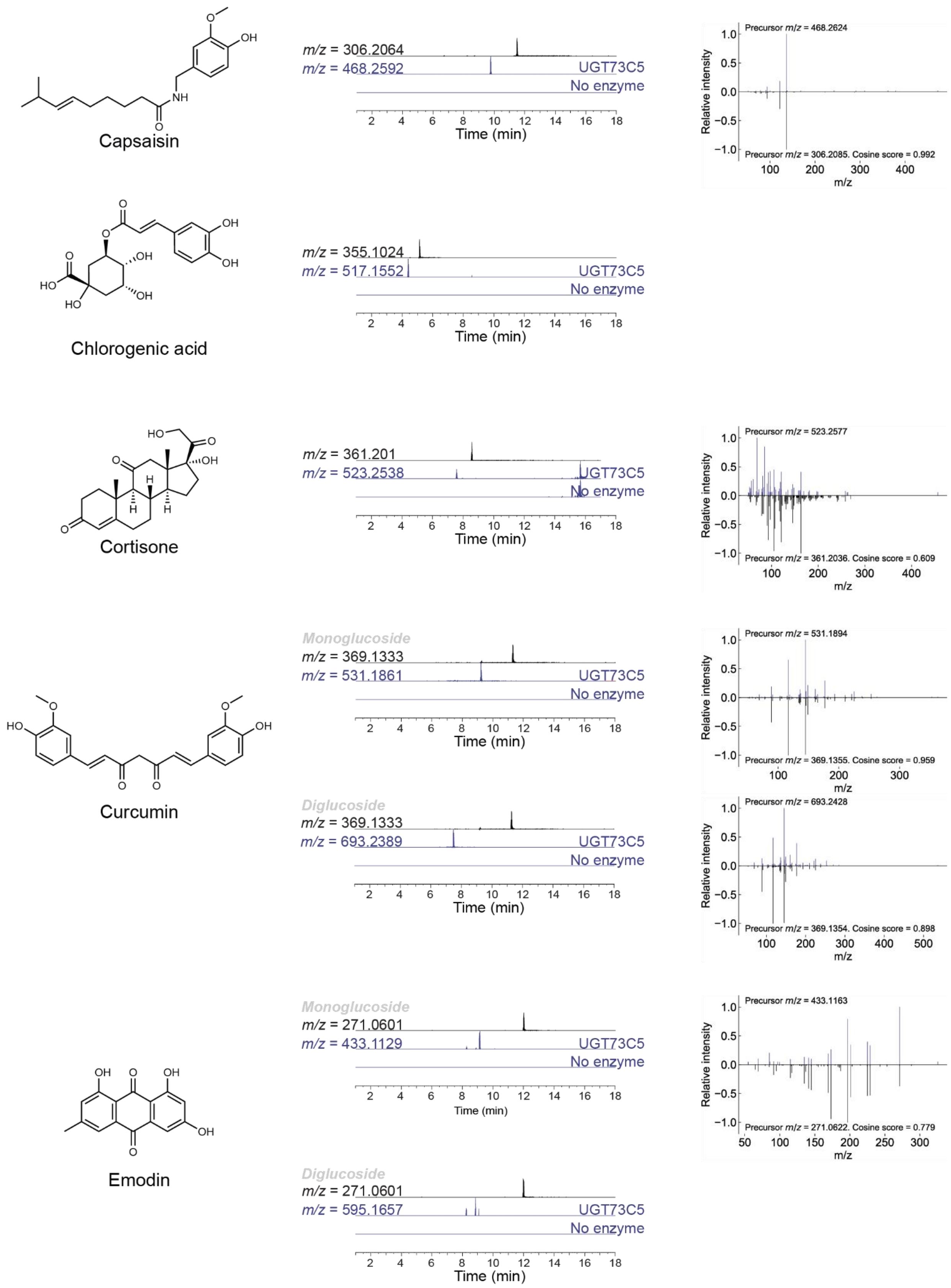

Supplementary Figure 14. Glycosylation products of capsaicin; chlorogenic acid; cortisone; curcumin; and emodin by purified UGT73C5. See Supplementary Fig. 13 legend for details.

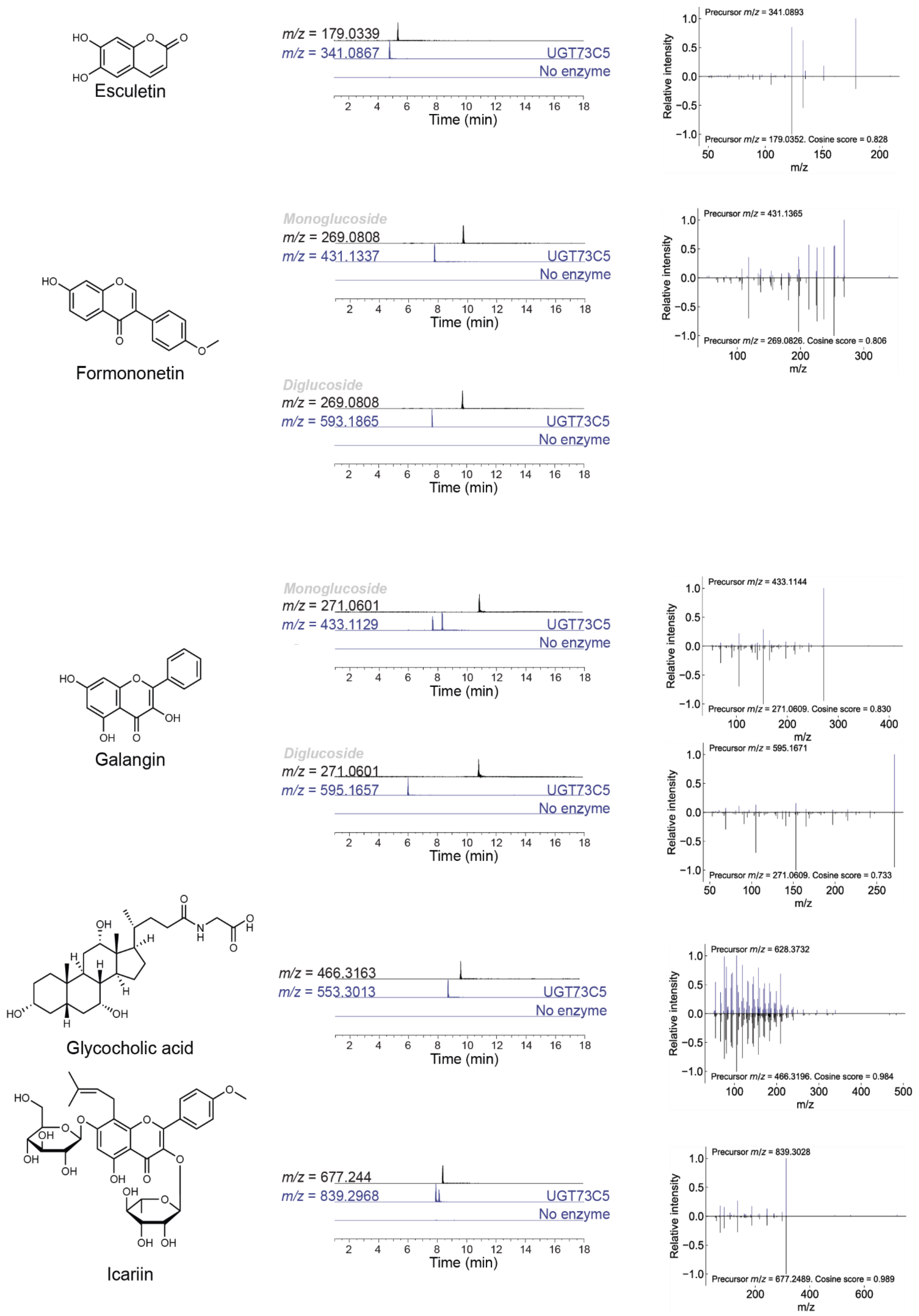

Supplementary Figure 15. Glycosylation products of esculetin; formononetin; galangin; glycocholic acid; and icariin by purified UGT73C5. See Supplementary Fig. 13 legend for details.

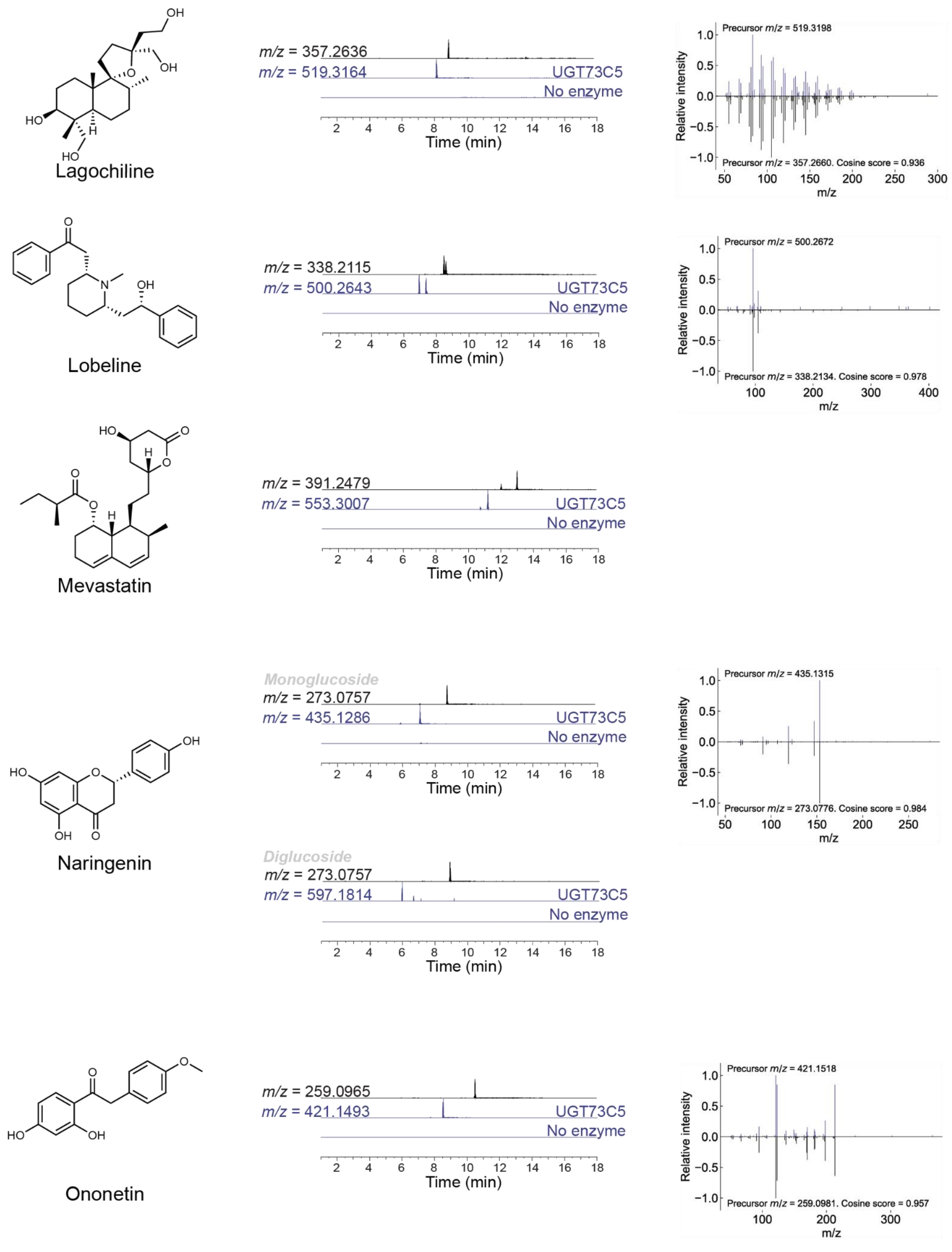

Supplementary Figure 16. Glycosylation products of lagochilin; lobeline; mevastatin; naringenin; and ononetin by purified UGT73C5. See Supplementary Fig. 13 legend for details.

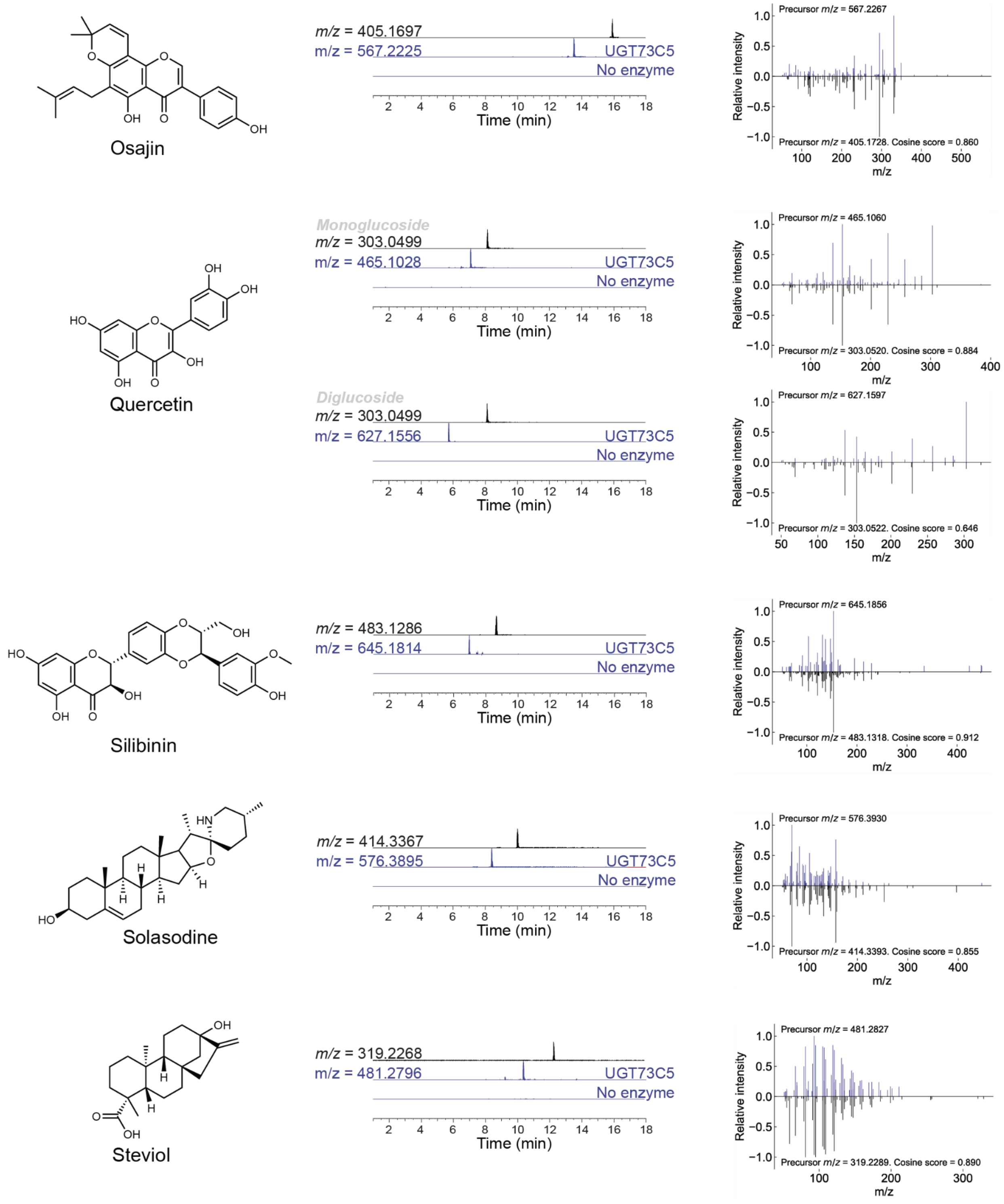

Supplementary Figure 17. Glycosylation products of osajin; quercetin; silibinin; solasodine; and steviol by purified UGT73C5. See Supplementary Fig. 13 legend for details.

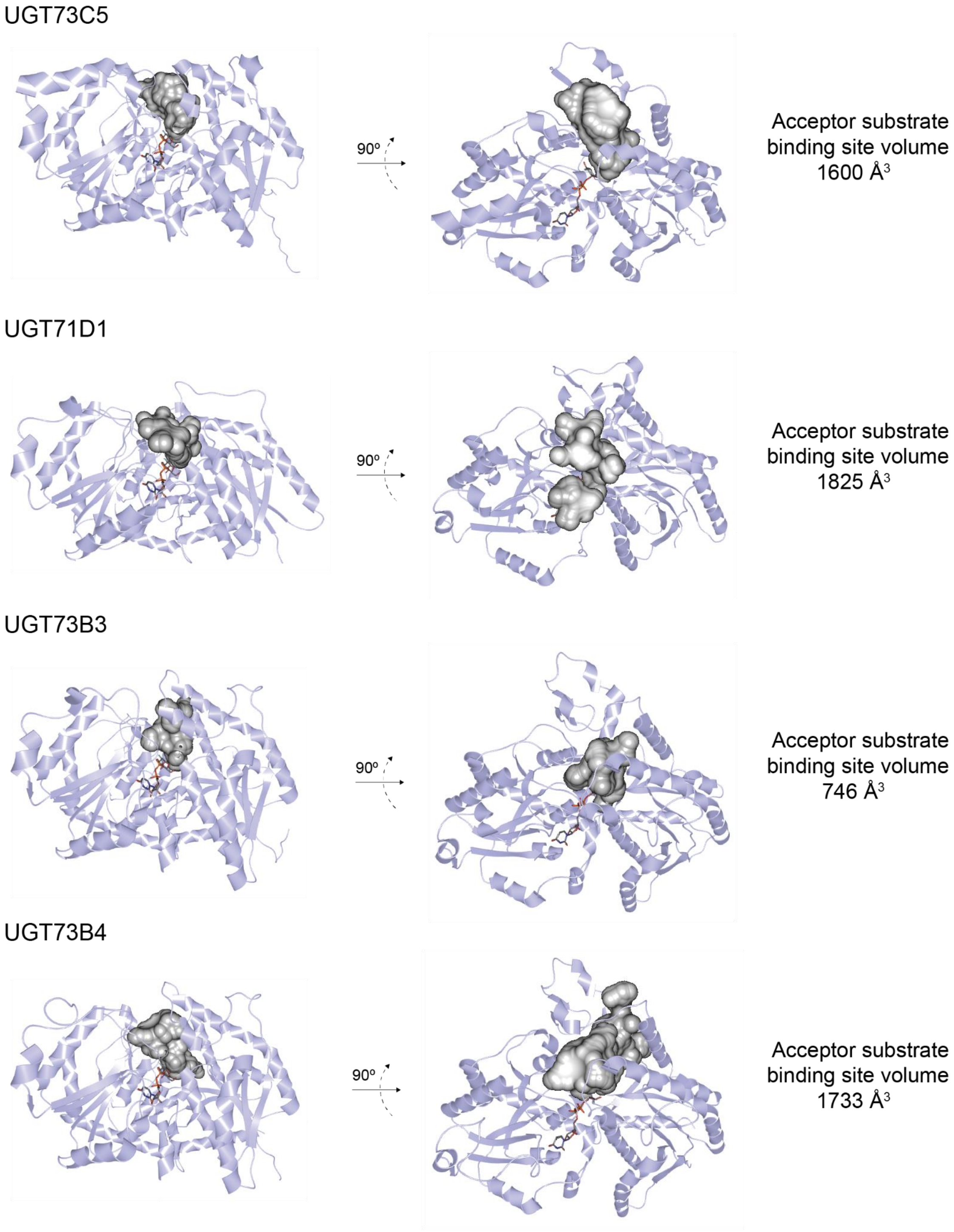

Supplementary Figure 18. Sugar acceptor binding pockets of the most promiscuous enzymes in the lysate screen. The volume of the putative sugar acceptor binding pocket of four most promiscuous enzymes in the lysate screen was measured from their structural models downloaded from AlphaFold Protein Structure Database superimposed with UDP-2-deoxy-2-fluoroglucose using Caver Analyst 2.0[59] using probe sizes of 1.8 and 4.0 Å. All four enzymes show expansive binding pockets easily accessible from the bulk solvent.

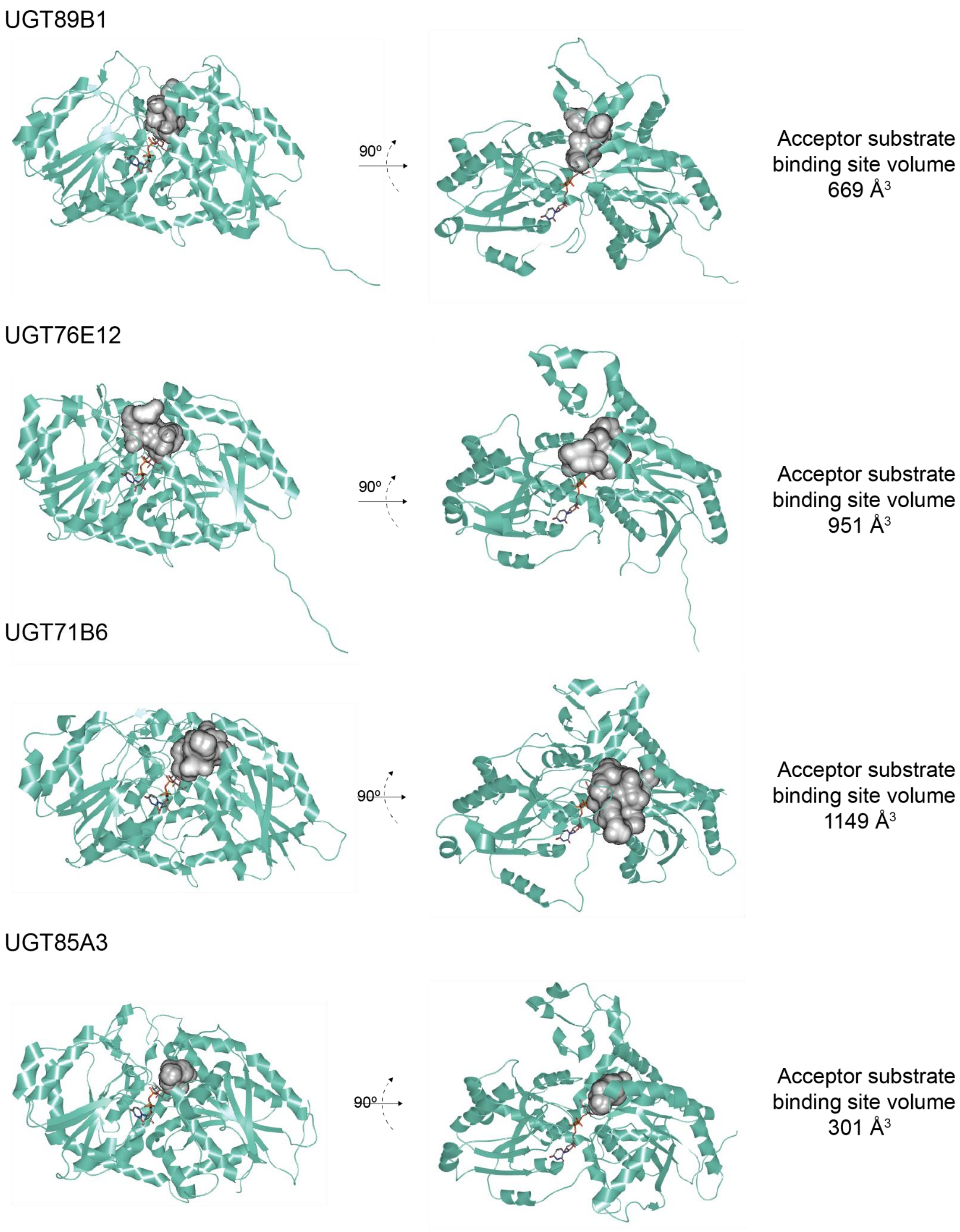

Supplementary Figure 19. Sugar acceptor binding pockets of the least promiscuous enzymes in the lysate screen. The volume of the putative sugar acceptor binding pocket of four most promiscuous enzymes in the lysate screen was measured from their structural models downloaded from AlphaFold Protein Structure Database superimposed with UDP-2-deoxy-2-fluoroglucose using Caver Analyst 2.0[59] using probe sizes of 1.8 and 4.0 Å. The four enzymes show binding pockets of varying sizes and accessibility levels.

a

b

c

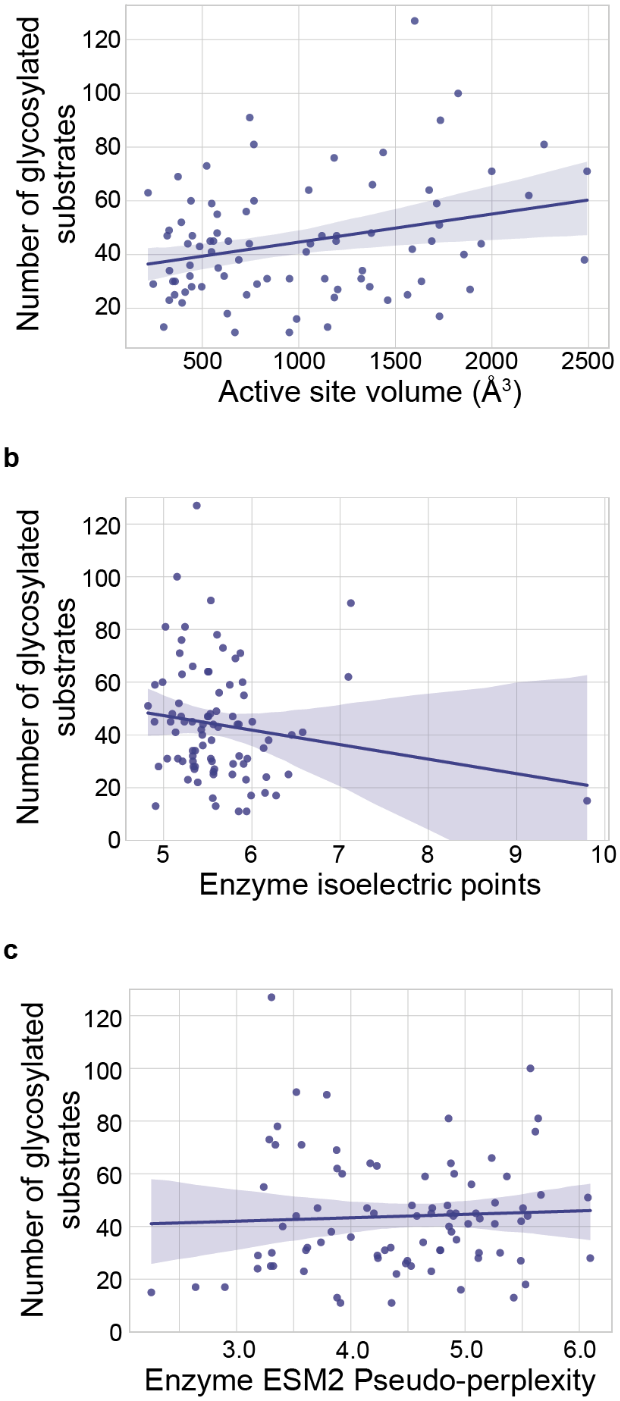

Supplementary Figure 20. Relationships between observed promiscuity and physical characteristics of enzymes. Each enzyme's active site volume (a), isoelectric point (b), and pseudo-perplexity (c) are plotted against the number of substrates glycosylated by that enzyme, which is assumed as a proxy for enzyme promiscuity. A weak positive correlation is observed between the volume of putative sugar acceptor substrate binding size measured with Caver Analyst 2.0[59] using probe sizes of 1.8 and 4.0 Å and enzyme promiscuity. On the other hand, no correlation was observed between isoelectric point and pseudo-perplexity, which are proxies for enzyme stability and expression levels, and enzyme promiscuity.

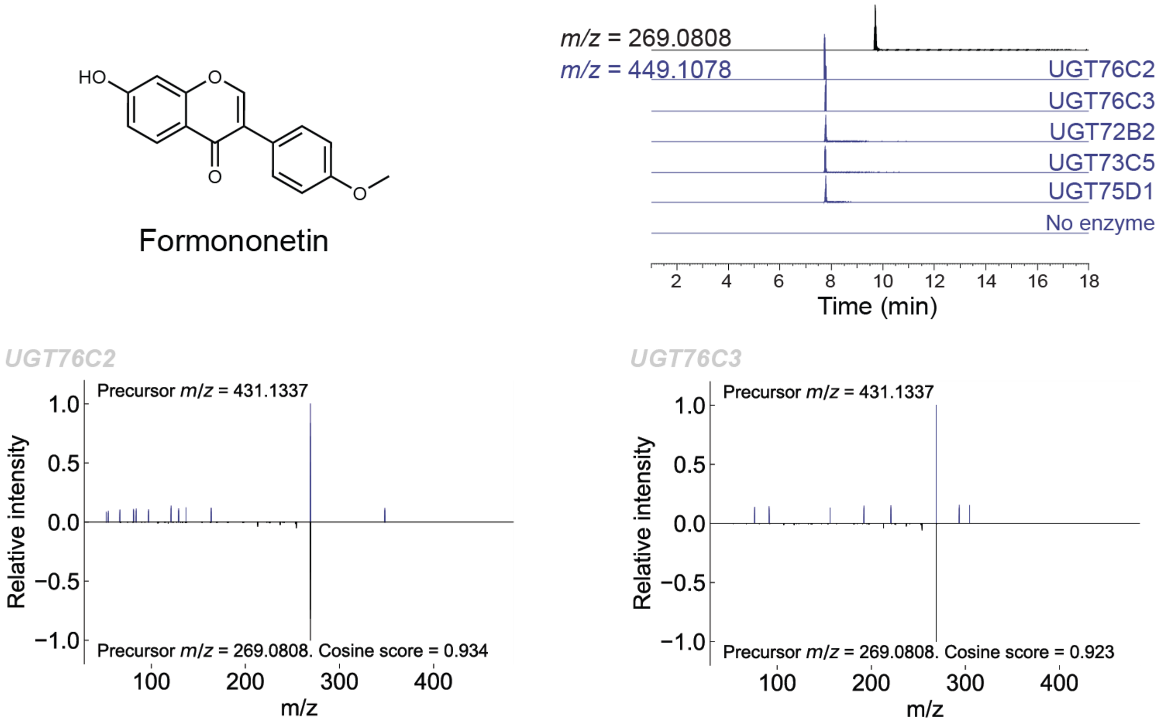

Supplementary Figure 21. *O*-glycosylation activity of family 1 GT enzymes with non-canonical Cys-Asp catalytic dyad. Extracted ion chromatograms of glycosylation products (purple) of formononetin produced by UGT76C2 and UGT76C3 (top right) show that *O*-glycosylation reactions can be mediated by family 1 GTs with non-canonical Cys-Asp catalytic dyad. Mass signals produced by UGT76C2 and UGT76C3 are weak but match those produced by other enzymes with canonical catalytic dyad in this study (*ie*. UGT72B2, UGT73C5, and UGT75D1; top right) with regards to exact mass, fragmentation pattern, and retention time. The extracted ion chromatogram of formononetin substrate (black) shows that glycoside's retention time shifts forward relative to its aglycone counterpart. Corresponding *m/z* values to [M+H]+ are indicated to the left of the chromatograms. Mirror plots corresponding to glycosylation products of formononetin produced by UGT76C2 (bottom left) and UGT76C3 (bottom right) show the comparison between an experimental MSMS spectrum (purple, top) and its matching reference spectrum (black, bottom) along with cosine scores.

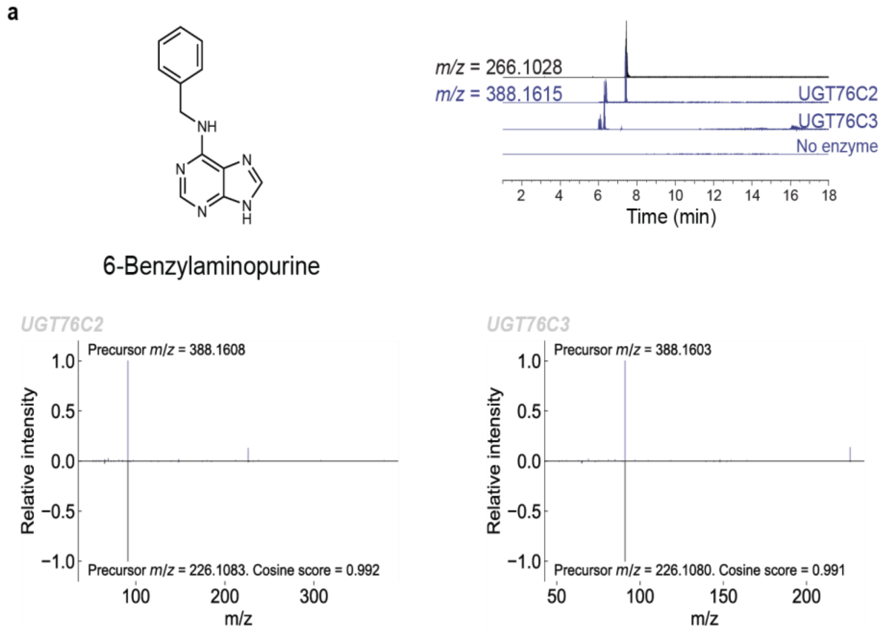

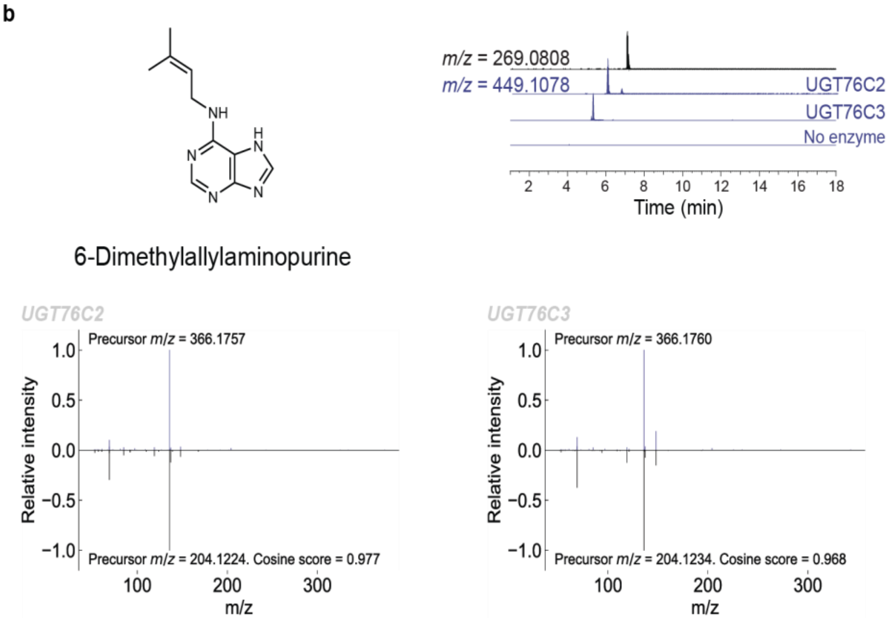

Supplementary Figure 22. *N*-glycosylation activity of family 1 GT enzymes with non-canonical Cys-Asp catalytic dyad. Extracted ion chromatograms of glycosylation products (purple) of 6-benzylaminopurine (a) and 6-dimethylallylaminopurine (b) produced by UGT76C2 and UGT76C3 (top right) show that *N*-glycosylation reactions can be mediated by family 1 GTs with non-canonical Cys-Asp catalytic dyad. Extracted ion chromatograms of the substrates (black) show that glycosides' retention times shift forward relative to their aglycone counterpart. Corresponding *m/z* values to [M+H]+ are indicated to the left of the chromatograms. Mirror plots corresponding to glycosylation products produced by UGT76C2 (bottom left) and UGT76C3 (bottom right) show the comparison between an experimental MSMS spectrum (purple, top) and its matching reference spectrum (black, bottom) along with cosine scores.

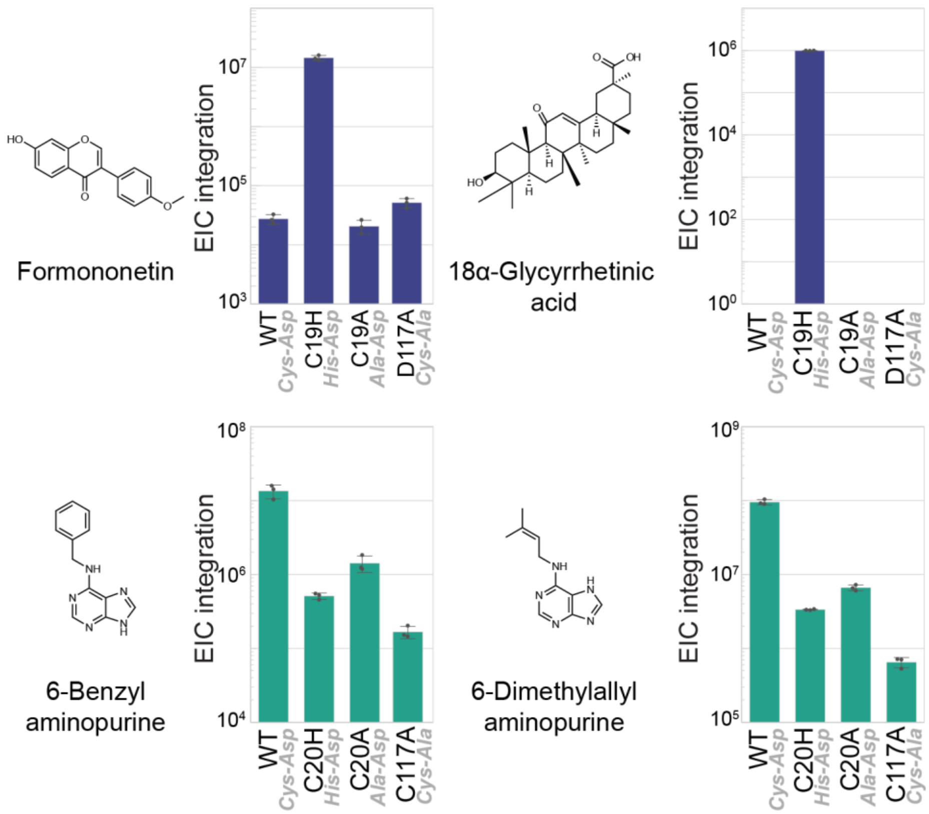

Supplementary Figure 23. *O*- and *N*-glycosylation activities of UGT76C3 and its active site variants. Integrations of extracted ion chromatograms of glycosylation products of formononetin (top left), 18α-glycyrrhetinic acid (top right), 6-benzylaminopurine (bottom left), and 6-dimethylallylaminopurine (bottom right) produced by UGT76C3 wild-type and catalytic dyad variants show that *O*-glycosylation reactions (blue) are favored by UGT76C3 C19H while *N*-glycosylation reactions (green) are favored by UGT76C3 WT. The catalytic dyad in each enzyme variant is shown in gray italics. Structures of sugar acceptor substrates are shown to the left of bar plots. Data are mean ± s.d. of three technical replicates.

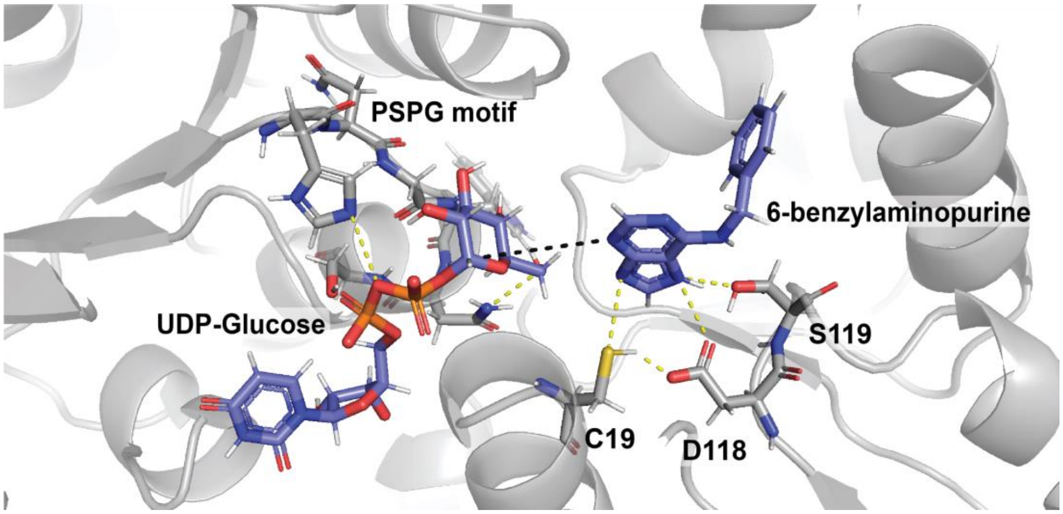

Supplementary Figure 24. Proposed substrate binding configuration in *N*-glycosylation reaction facilitated by family 1 GT enzymes with non-canonical Cys-Asp catalytic dyad. Docking experiments show that Asp118 may help align the sugar acceptor substrate 6-benzylaminopurine in a catalytically competent orientation by forming interactions with the nitrogen atoms of the purine ring. In this proposed binding mode, the cysteine residue in the active site leaves an open space where *N*-glycosylation reaction can occur, whereas a histidine residue at the same position may sterically hinder the reaction.
