# Supplementary material for: A substrate-multiplexed platform for profiling enzymatic potential of plant family 1 glycosyltransferases
Source: Nat Commun. 2025 Jul 10;16:6366. doi: 10.1038/s41467-025-61530-6 (PMC12246196; doi:10.1038/s41467-025-61530-6)
Supplement: Supplementary file 3 — Description of Additional Supplementary Files [file 41467_2025_61530_MOESM3_ESM.pdf]

Description of Additional Supplementary Files

File Name: Supplementary Data 1
Description: Family 1 GT enzymes from Arabidopsis included in this study

File Name: Supplementary Data 2
Description: Sugar acceptor candidate substrates included in this study

File Name: Supplementary Data 3
Description: Lysate-based screening results using cosine score threshold of 0.85

File Name: Supplementary Data 4
Description: Lysate-based screening results using cosine score threshold of 0.75

File Name: Supplementary Data 5
Description: Lysate-based screening results using cosine score threshold of 0.80

File Name: Supplementary Data 6
Description: Lysate-based screening results using cosine score threshold of 0.90
