# Supplementary material for: A substrate-multiplexed platform for profiling enzymatic potential of plant family 1 glycosyltransferases
Source: Nat Commun. 2025 Jul 10;16:6366. doi: 10.1038/s41467-025-61530-6 (PMC12246196; doi:10.1038/s41467-025-61530-6)
Supplement: Supplementary file 10 — Reporting Summary [file 41467_2025_61530_MOESM10_ESM.pdf]

nature portfolio

Corresponding author(s): Patrick Shih

Last updated by author(s): Jun 9, 2025

Reporting Summary

Nature Portfolio wishes to improve the reproducibility of the work that we publish. This form provides structure for consistency and transparency in reporting. For further information on Nature Portfolio policies, see our Editorial Policies and the Editorial Policy Checklist.

Statistics

For all statistical analyses, confirm that the following items are present in the figure legend, table legend, main text, or Methods section.

n/a	Confirmed	
☐	☒	The exact sample size (*n*) for each experimental group/condition, given as a discrete number and unit of measurement
☐	☒	A statement on whether measurements were taken from distinct samples or whether the same sample was measured repeatedly
☒	☐	The statistical test(s) used AND whether they are one- or two-sided<br>*Only common tests should be described solely by name; describe more complex techniques in the Methods section.*
☒	☐	A description of all covariates tested
☒	☐	A description of any assumptions or corrections, such as tests of normality and adjustment for multiple comparisons
☐	☒	A full description of the statistical parameters including central tendency (e.g. means) or other basic estimates (e.g. regression coefficient) AND variation (e.g. standard deviation) or associated estimates of uncertainty (e.g. confidence intervals)
☒	☐	For null hypothesis testing, the test statistic (e.g. *F*, *t*, *r*) with confidence intervals, effect sizes, degrees of freedom and *P* value noted<br>*Give P values as exact values whenever suitable.*
☒	☐	For Bayesian analysis, information on the choice of priors and Markov chain Monte Carlo settings
☒	☐	For hierarchical and complex designs, identification of the appropriate level for tests and full reporting of outcomes
☒	☐	Estimates of effect sizes (e.g. Cohen's *d*, Pearson's *r*), indicating how they were calculated

Our web collection on statistics for biologists contains articles on many of the points above.

Software and code

Policy information about availability of computer code

Data collection	Mass spectra were collected using Thermo Fisher XCalibur 4.3.
Data analysis	Mass spectra were analyzed using Thermo Fisher FreeStyle 1.6 and custom codes. Protein structural models retrieved from AlphaFold Protein Structure Database were analyzed using Caver Analyst 2.0 and AutoDock Vina 1.2.0, and visualized using PyMOL 2.5.5.

For manuscripts utilizing custom algorithms or software that are central to the research but not yet described in published literature, software must be made available to editors and reviewers. We strongly encourage code deposition in a community repository (e.g. GitHub). See the Nature Portfolio guidelines for submitting code & software for further information.

Data

Policy information about availability of data

All manuscripts must include a data availability statement. This statement should provide the following information, where applicable:

- Accession codes, unique identifiers, or web links for publicly available datasets
- A description of any restrictions on data availability
- For clinical datasets or third party data, please ensure that the statement adheres to our policy

The lysate-based screening data are provided as Supplementary Data Files. Numerical values shown in Figures are provided with this paper as a Source Data file or at https://github.com/vinbl/gtscreen/tree/main. Raw mass spectrometry data files are available at DOI 10.5281/zenodo.15670422. All nucleotide sequences used in this manuscript are available at https://public-registry.jbei.org

Research involving human participants, their data, or biological material

Policy information about studies with human participants or human data. See also policy information about sex, gender (identity/presentation), and sexual orientation and race, ethnicity and racism.

Reporting on sex and gender	NA
Reporting on race, ethnicity, or other socially relevant groupings	NA
Population characteristics	NA
Recruitment	NA
Ethics oversight	NA

Note that full information on the approval of the study protocol must also be provided in the manuscript.

Field-specific reporting

Please select the one below that is the best fit for your research. If you are not sure, read the appropriate sections before making your selection.

☒ Life sciences ☐ Behavioural & social sciences ☐ Ecological, evolutionary & environmental sciences

For a reference copy of the document with all sections, see nature.com/documents/nr-reporting-summary-flat.pdf

Life sciences study design

All studies must disclose on these points even when the disclosure is negative.

Sample size	No statistical analyses were used to pre-determine sample size. For the lysate-based screening data, data shown is of one biological replicate. For all other chromatograms and mass spectra, data shown is representative of at least three biological replicates. The sample size was chosen to determine reproducibility based on experience of variability in these assays. For enzyme assays with quantitative data, three technical replicates were used to determine mean and standard deviation values, which we state where relevant.
Data exclusions	No data was excluded from the analyses.
Replication	For the lysate-based screening data, one biological replicate was performed. No replication was carried out due to practical limitations. For all other data showing representative chromatograms and mass spectra, at least three biological replicates were performed. All attempts at replication were successful.
Randomization	No data were randomized since it is not applicable to our sets of experiments. Randomization does not apply because experimental data are measurements of protein activity.
Blinding	There was no blinding as it is not applicable for our sets of experiments. Blinding does not apply because experimental data are measurements of protein activity and do not involve human subjects.

Reporting for specific materials, systems and methods

We require information from authors about some types of materials, experimental systems and methods used in many studies. Here, indicate whether each material, system or method listed is relevant to your study. If you are not sure if a list item applies to your research, read the appropriate section before selecting a response.

Materials & experimental systems

n/a	Involved in the study	
☒	☐	Antibodies
☒	☐	Eukaryotic cell lines
☒	☐	Palaeontology and archaeology
☒	☐	Animals and other organisms
☒	☐	Clinical data
☒	☐	Dual use research of concern
☒	☐	Plants

Methods

n/a	Involved in the study	
☒	☐	ChIP-seq
☒	☐	Flow cytometry
☒	☐	MRI-based neuroimaging

Plants

Seed stocks	NA
Novel plant genotypes	NA
Authentication	NA
